# WEEE polymers valorization, its use as fuel in the gasification process and revaluation of the inert by-products obtained: Sustainable mortars as a solution

**DOI:** 10.1016/j.heliyon.2023.e20194

**Published:** 2023-09-15

**Authors:** Daniel Díaz-Perete, Manuel Jesús Hermoso-Orzáez, Julio Terrados-Cepeda, Pedro Silva-Romano, Cristina Martin-Doñate

**Affiliations:** a Department of Graphic Engineering, Design and Projects, University of Jaén, 23071, Jaén, Spain; b Centre for Advanced Studies in Energy and Environment, University of Jaén, 23071, Jaén, Spain; c VALORIZA - Research Center for Endogenous Resource Valorization, Polytechnic Institute of Portalegre, Portugal IP Portalegre - Polytechnic Institute of Portalegre, Portalegre, Portugal

**Keywords:** Polymer valorization methodology, Polymer waste, Gasification waste, Waste-to-By-product, Sustainability, WEEE, Mechanical tests, Industry-to-Building, Inert-valorization

## Abstract

The global production of polymer materials has exploded in the last few decades. Their mechanical properties, erosion and corrosion resistance, good performance as insulation materials, and their ease and flexibility of manufacturing have made polymers one of the most widely used materials in the industry and in daily life. Several institutions and governments are beginning to raise serious environmental and ecological concerns with international impact soon, due to the increasing level of polymer production, which does not seem to be slowing down. It is necessary for the scientific community to make efforts in the development and evaluation of new methodologies to enable the inclusion of these types of materials in the circular economy of various production sectors. This is important in order to reduce the ecological impact caused by the current global production level of polymers. One of the most used methods for the recovery of polymeric materials is energy valorization through thermochemical processes. An example of this is thermal gasification using fuels composed of biomass and a mixture of polymeric waste from electrical and electronic equipment (WEEE). Through this thermochemical process, high-energy value synthesis gas, with a high concentration of hydrogen, is obtained on one hand, while waste products in the form of chars, ashes and slag are generated on the other hand. This manuscript presents a detailed study methodology that begins with chemical analysis of the raw material and includes subsequent analysis of mechanical results for the revaluation of these residual inert by-products, using them as partial substitutes in cement clinker to produce building mortars. This described methodology influences directly in the LCC (Life Cycle Costing) of final designed products in plastic and extend material life cycle Plastic materials are here to stay, so the study and optimization of polymer waste recovery processes are vital in achieving the Sustainable Development Goals (SDGs) set by the European Union in terms of efficiency and sustainability. It is also the only possible way to create an environmentally sustainable future world for future generations. After applying the described methodology, the mechanical test results show that the modified mortars exhibit established behaviour during the hardening time and similar strength growth compared to commercial mortars. The maximum mechanical strengths achieved, including compressive and flexural strength, make modified mortars a viable choice for several applications in the civil engineering sector.

## 1 Introduction

Polymer materials are practically ubiquitous in the daily activities of the average human being. Most objects with which people interact throughout the day are composed of or include polymer materials to varying degrees [1]. Plastics have become definitive materials for many production sectors due to their cost, ease of manufacture, and diverse properties. Polymers are already present in a wide range of products, including automotive vehicles, electronic components such as computers, laptops, and smartphones, various types of clothing, and they dominate the food industry as principal packaging materials [2]. Materials technology in the field of polymers has evolved rapidly in tandem with the increasing volume of their production [3,4]. With each advancement, polymers offer better performance in terms of mechanical, physical, and thermal insulation properties. Injection molding is the primary manufacturing method used for polymers, and researchers and companies are testing and producing the use of plastics in combination with other materials to achieve higher performance in mechanical, physical, or chemical properties, with promising results [5,6]. The evolution of manufacturing techniques in Industry 4.0, such as additive manufacturing, relies on the availability of these easily manufacturable materials and is based on automation and robotics. Polymers are undoubtedly the present and future of modern industry, and as a result, international plastic production levels have already reached alarming levels, leading to a departure from the desired sustainability and proper maintenance of ecosystems worldwide. These waste materials are beginning to accumulate in various locations around the inhabited world and are coexisting with marine creatures in our oceans as well [7,8].

We are faced with a serious global challenge. Although the typology and composition of polymers vary greatly in terms of their physical and chemical properties, four main methods of recovery are described in scientific literature, summarized as follows.

• Primary recovery or mechanical processing, elaborating the same product as the original polymer was [9,10].
• Secondary recovery or downgrading recovery, where less quality plastic parts are recovered through mechanical processing from used polymer parts [10,11].
• Tertiary recovery or chemical recovery, to recover petrochemical components from polymers used parts [[12], [13], [14]].
• Energy recovery, through incineration a fraction of energy used in polymer part production is recovered in form of heat.[12] [15].

This manuscript is based on the fourth main method of polymer waste recovery mentioned above. The described process in this research begins with thermal gasification as an energy recovery process. Gasification is a variation of this energy recovery method, involving a thermo-chemical transformation process that produces a high-value energetic gas called synthesis gas or syngas. Syngas is generated through the combustion of the raw material, which acts as fuel in the gasifier. In this case, the fuel is a compound consisting of a mixture of WEEE polymer materials and biomass [16]. Therefore, syngas is the primary product resulting from this thermo-chemical process, but it also generates residual products. These waste products typically take the form of ash and slag, and their chemical composition depends on the type of fuel used and its composition. The methodology presented in this manuscript considers the residual products obtained during the gasification process as by-products and proposes a viable option for their integration into the circular economy of the construction sector by incorporating them into mortars. This approach offers a new use for the produced ash and extends the service life cycle of the polymer materials contained in WEEE.

The ashes, which have undergone various chemical and physical treatments as explained in the materials and methods section of this document, replace 10% of the total volume of mortar clinker [17]. The thermal gasification of biomass mixed in proportions of up to 20% with plastic and rubber waste yields highly energy-efficient syngas and enables the recovery of waste that would otherwise be sent to landfills [18].

In terms of the life cycle costing (LCC) of projects and plastic products, the utilization of by-products after energy recovery, as proposed in this methodology, represents a novel and optimistic paradigm. From an environmental impact perspective, it has a positive impact, but it can also influence the selection of different life cycle inventories (LCI) in projects based on the resulting by-product properties and the requirements of downstream industries. This creates new possibilities for circular business models to implement the Sustainable Development Goals (SDGs) promoted by the European Union institutions [19]. The LCC is understood as the economic support of the LCA and complements it. It could be defined as the historical and current practice of many governments and companies and is based on a purely economic evaluation. It quantifies the production costs of the resources consumed and costs associated with the different phases of the design, production, management and removal of the project [20]. In recent scientific literature has been exposed than Life Cycle Assessment (LCA) and Life Cycle Costing (LCC) tools are applied to estimate the environmental and economic life-cycle profile of the synthetic biofuels, for example, when potential improvement actions are implemented [21,22].

**Fig. 1:**
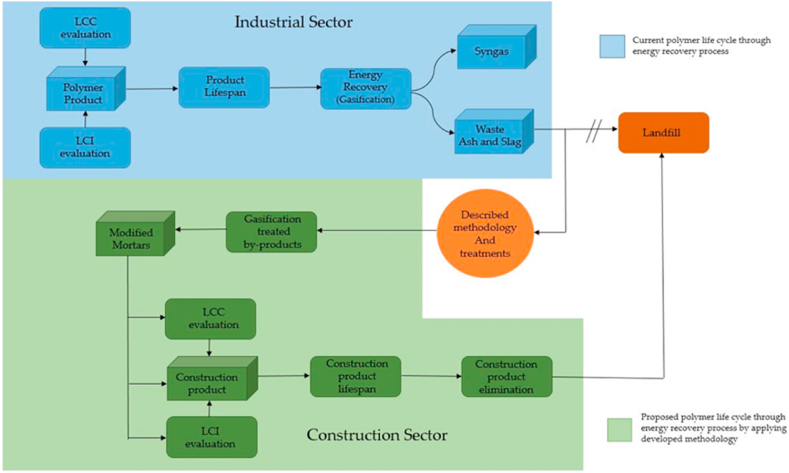
Diagram flow of life cycle extension by applying methodology described in manuscript to WEEE polymers.

In summary, when by-products are considered valuable raw materials instead of inert waste, there is a direct reduction in the LCC of the designed project. In other words, the initial products are potentially more economically sustainable than if the residues obtained after energy recovery processes were sent directly to landfills. Fig. 1 illustrates the flow diagram of the objectives accomplished by the proposed methodology.

**Fig. 2:**
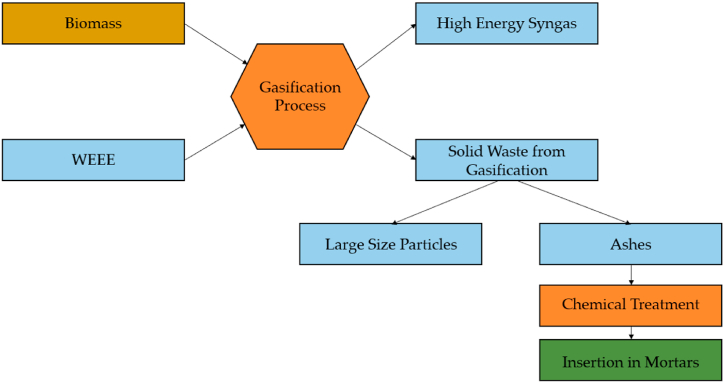
Flow diagram of the general process for obtaining ashes for mortars.

The manuscript also includes a comparison of the mechanical behaviour, including flexural strength and uniaxial compressive strength, between modified mortars and standard building mortars. The aim of this comparison is to verify the proposed polymer recovery methodology, which seeks to incorporate these materials into the circular economy of the construction sector. The modifications applied to the mortars involve partially substituting the binder, which is typically Portland concrete clinker, with ashes obtained as a product of the gasification process [23]. However, the ashes produced after the gasification processes (see Fig. 2) present certain challenges in terms of their integration into the industrial circular economy. In order to address this issue, the research proposes the revalorization of these inert by-products in the civil engineering field by incorporating them into mortars as partial substitutes for Portland clinker. The results section of this paper demonstrates that these modified mortars exhibit acceptable mechanical behaviour [24,25].

All specimens included in this work, including modified mortars and common building mortar, were created using the same method. The specimens have been made in situ, therefore, in order to do a correct comparison and interpretation of the obtained results in the experimental mechanical tests, standard mortar made of Portland concrete have been taken by reference. [26].

In the absence of hardening retarders, the European UNE-EN 1015–11:2020 [27,28] standard requires that mortar specimens be tested 28 days following manufacture. This document covers experimental mechanical testing of the first 7 days of the mortars to evaluate the progression of their flexural and uniaxial compression strength during their hardening time, in addition to 28 days of experimental mechanical tests.

Obtained results of mentioned mechanical tests are presented according current UNE-EN 196–1:2018 [29] standard in results and discussions section of this manuscript. It is also included in the paper, a comparison between mechanical results obtained by using the presented methodology and other studies of modified mortars to quantify numerically the applicability of this recovery industrial by-products method and its efficiency in the construction sector.

The presented research has developed a methodology on the recovery, reuse and increase of WEEE life cycle used in combination with biomass as fuel into a gasification process. As a result of this process, syngas is obtained as an energy resource. Furthermore, in a second step the treatments suffered by resultant ashes from gasification are presented. Including the chlorine levels reduction of the by-products, to obtain mechanically stable mortars after the inclusion of above-mentioned ashes used as partial clinker substitutes of Portland cement. It is demonstrated the capacity of recovery of these industrial waste from polymer materials, including them into the construction sector circular economy in form of modified mortars after being checked qualitatively through mechanical tests which indicate the stable properties for their use in construction products.

## 2 Materials and methods

In order to properly conduct the experimental mechanical tests, a total of 18 specimens were manufactured. Among these, 6 specimens were made using standard Portland concrete, while the remaining 12 specimens were modified. The modifications applied to the latter group of specimens involved a partial substitution of Portland clinker with fly ashes that were recovered through a gasification process.

### 2.1 Materials

#### 2.1.1 Cement

Used concrete in mortar specimens manufactured for this study is defined by UNE-EN 197–1 as CEM I 42.5 R. This classification indicate that used concrete is Portland cement, its resistance class is 42.5 and R of the definition means high initial resistance. Obeying UNE-EN 196 [29] standard, composition in mass of mentioned above concrete is composed of 100%–95% of clinker and between 0% and 5% of minorities components. Cement Portland clinker is obtained by a homogenous mixture of raw materials which content is formed by elements in form of oxides, CaO, SiO_2_, Al_2_O_3_, Fe_2_O_3_ and little quantities of minorities’ components [30,31].

Cement Portland clinker is a hydraulic material composed, at least two thirds of its mass, by calcium silicates, [3CaO·SiO_2_] and [2CaO·SiO_2_]. The rest of clinker contain aluminum, iron and other components minorities. Mass relationship (CaO)/(SiO) will not be less than two and the content of magnesium oxide (MgO) do not exceed 5% in its mass [30,31].

#### 2.1.2 Sand

**Fig. 3:**
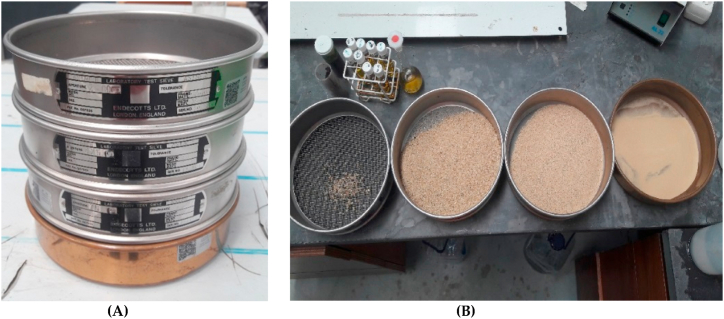
ASTM standard sieves **(A)**. Obtained results for sand sieve **(B)**.

**Fig. 4:**
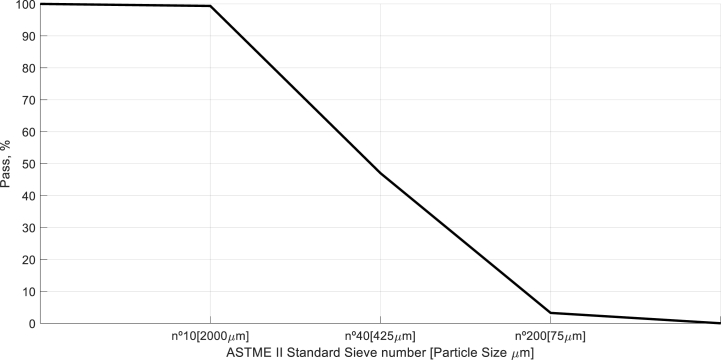
Particle size dispersion associated to sand used in mortar specimens of the manuscript.

Sand is another basic material for mortars manufacture. To elaborate the specimens of this manuscript washed sand was used. In soil mechanics, sand definition is the fraction of the soil who pass through nº10 ASTM sieve and do not pass through nº200 ASTM sieve [32]. Before the sand utilization in mortars, in order to identify quality of raw material and after the reception of the product, sand was sieved according ASTM standard to contrast its granulometry properties (see Fig. 3 a, b). Fig. 4, presents granulometric size dispersion of particles obtained for material sample, it shows sand has a balanced sized distribution granulometry with low distribution of fines. Numerically, the granulometric tests done to the obtained material expose 99.36% of particles which form the soil present lower diameter than 2000 μm, higher values of diameters are consider as gravel, and only 3.25% of particles forms the fine fraction of material, it means, particles count with diameters lower than 75 μm.

#### 2.1.3 Ashes

**Fig. 5:**
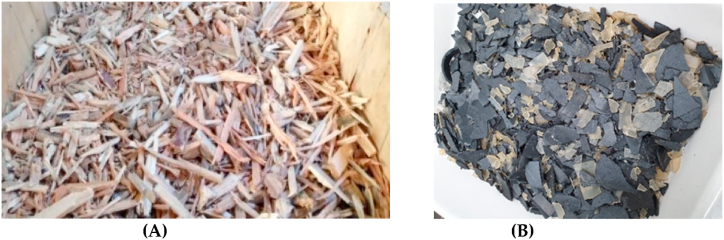
Raw material used for joint gasification in the reactor. At left **(A)**, pine-tree biomass. At right **(B)**, WEEE, polyethylene and polypropylene.

The study presents a comparison between commercial mortars, which are made of Portland cement, and modified mortars that involve a partial substitution of Portland clinker with ashes generated as waste from the gasification process [33]. Two different types of residual ashes were selected for this study, obtained from distinct gasification processes [34]. In the first gasification, raw material selected was a mixture between pine-tree biomass, 90%, and WEEE, 10%, formed principally by polyethylene and polypropylene, hereinafter ashes type I [23]. On the other hand, in second gasification raw material selected is formed by pine-tree biomass 100%, hereinafter ashes type II. (See Fig. 5 a, b).

After the gasification, before include ashes in the modified mortars design, a treatment of cleaning of inorganic elements and soluble mineral contained in ashes have done. The treatment consists of first washing the ashes in water and then in acetone [35]. Ashes produced during the gasification process are waste made by a mixture of biomass and plastic trash that do not react owing to oxygen excess throughout the process. Tars, soluble mineral, inert, and other organic compounds make up ashes. Ashes are largely composed of SiO2 and CaO, with minor amounts of Mg, Al, K, and P [36].

**Fig. 6:**
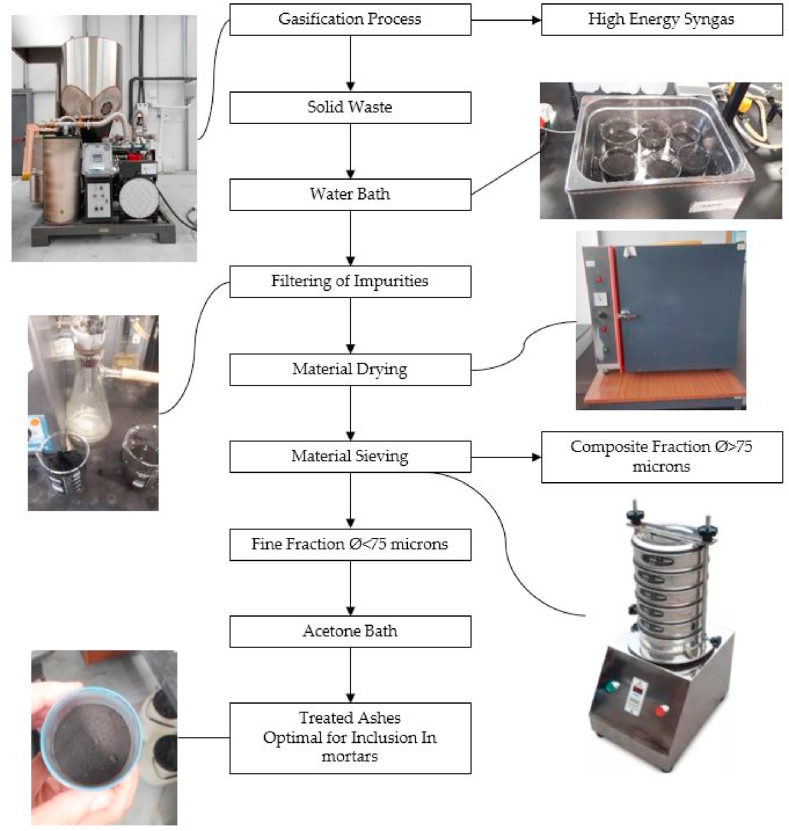
Diagram flow, laboratory process to obtain optimal ashes to include in mortars.

The initial stage in preparing necessary specimens is to separate fine granulometry from the remaining trash. This fine granulometry material is utilized to create the experimental tests mentioned in this publication. To begin the separation of fine granulometry, a coal compound created as a result of the gasification process was sieved. Following that, the gasification waste is washed with water, and the combination is subjected to an ultrasonic bath. This process takes 2 h, and the combination rests for the next 24 h [37]. After this time has passed, the mixture is filtered using Glass microfibre Discs 125 mm to remove any remaining water in the composition. The wash in water method is completed by placing the mixture in an oven for 12 h at 100 °C to remove moisture from the compound. Similarly, the mixture is washed in an acetone bath using the same techniques as the water bath. Finally, beginning material was sorted according to particle size using sieves of 2000 *m*, 850 *m*, 425 *m*, 180 *m*, 106 *m*, 75 *m*, and 75 *m*, respectively, utilizing Octagon digital CE equipment (See Fig. 6).

**Fig. 7:**
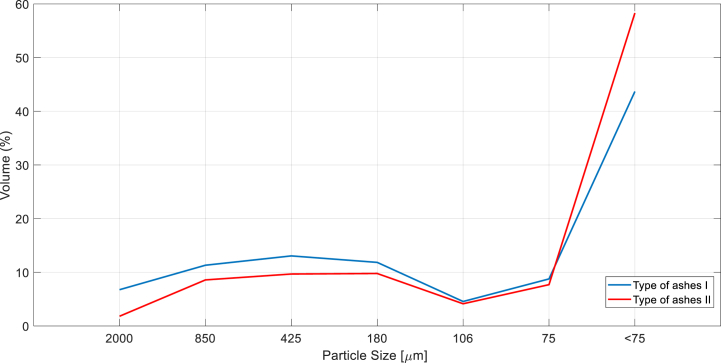
Particle size dispersion associated to recovered ashes from gasification to include in modified mortars.

When fine granulometry ashes have been separated of the rest of gasification residues (see Fig. 7), its chemical composition is verify through XRF analysis. Acetone bath purpose is to remove hydrocarbons, that are present in the particles surface, in order to avoid possible future corrosion in modified mortars specimens.

Previous heat treatments of biomass, to prepare ashes, and wash operations above-mentioned involve chemical changes in raw material. Table 1, shows concerning values to ashes chemical composition before and after water and acetone baths.

**Table 1:** Type I and Type II ashes chemical composition after suffering water and acetone bath.

Composition	Gross	Type II	Type II water	Type II acetone	Type I	Type I water	Type I acetone
**Zn**	0.00	0.36	0.63	0.59	0.42	0.39	0.38
**Cu**	0.13	0.35	0.32	0.23	0.13	0.14	0.14
**Ni**	0.00	0.18	0.19	0.34	0.00	0.06	0.05
**Fe**	0.00	7.13	10.40	17.82	5.22	7.63	11.18
**Mn**	0.00	0.28	0.34	0.32	0.06	0.16	0.11
**Cr**	0.00	0.71	0.65	1.29	0.03	0.16	0.14
**V**	0.28	0.07	0.05	0.05	0.03	0.05	0.04
**Ti**	72.75	8.16	0.87	0.88	0.41	0.68	0.69
**Sc**	0.00	0.14	0.06	0.08	0.07	0.10	0.09
**Ca**	12.47	67.89	70.92	64.35	36.34	53.48	51.38
**K**	2.16	6.61	4.64	4.61	36.62	20.84	19.72
**S**	0.00	1.13	1.72	1.36	3.24	2.15	1.71
**P**	0.00	0.29	0.57	0.37	1.22	2.47	2.44
**Si**	0.00	4.07	6.22	5.72	8.07	8.36	8.91
**Cl**	11.63	2.40	2.07	1.72	7.86	3.09	2.77

The results in Table 1 show that ash treatments lowered Ti, Ca, K, and Cl concentrations [38]. High levels of chlorine and potassium in the sample can encourage deposit development, mortar degradation, and even limit the incorporation and use of ashes [39]. The chlorine content of type 2 ashes was reduced by 82.20% and 84.80% after water and acetone baths, respectively, whereas the chlorine content of type 1 ashes was reduced by 73.43% and 76.18% [40].

Fly ash, slag, metakaolin, blast furnace slag, or ashes from rice trash are high aluminosilicated materials that can be used as binders in mortars and concrete [41]. Binder ingredients play a significant role on the mechanical properties of final products, in this case mortars, after the hardening process [42]. The primary constituents of the source material are SiO2, Al2O3, Fe2O3, Na2O, K2O, and CaO. The given composition is 90% SiO2, Al2O3, CaO, and Fe2O3, with a relatively low presence of other oxides [41]. These oxides have a significant impact on the mechanical characteristics of final products [43]. Table 2 displays the chemical composition of the resulting ashes attending its oxides.

**Table 2:** Chemical composition attending to oxides of resultant ashes.

Chemical Composition of Ashes	Type II	Type II water	Type II acetone	Type I	Type I water	Type I acetone
**CaO**	94.983	99.214	90.021	50.838	74.82	71.874
**Al**_**2**_**O**_**3**_	–	–	–	–	–	–
**MgO**	–	–	–	–	–	–
**SiO**_**2**_	8.698	13.309	12.228	17.259	17.883	19.056
**Na**_**2**_**O**	–	–	–	–	–	–
**SO**_**3**_	2.81	4.294	3.384	8.082	5.374	4.274
**P**_**2**_**O**_**5**_	1.34	2.612	1.699	0.00	11.326	11.199
**K**_**2**_**O**	7.964	5.594	5.554	44.127	25.11	23.761
**TiO**_**2**_	13.605	1.449	1.461	0.677	1.136	1.148
**MnO**	0.21	0.25	0.239	0.905	0.119	0.082
**Fe**_**2**_**O**_**3**_	10.197	14.873	25.488	7.458	10.906	15.983

### 2.2 Specimens manufacturing process

**Fig. 8:**
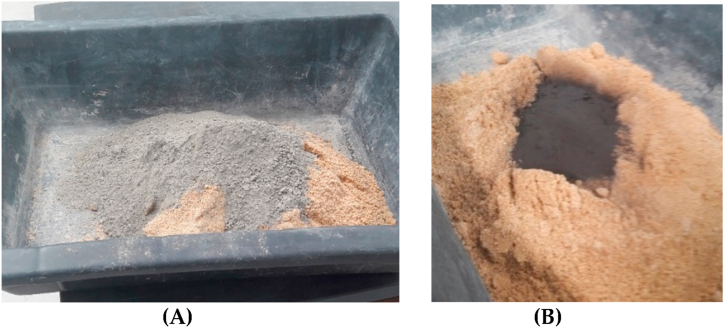
Examples of the elaboration of mortar M1 **(A)** and mortar M2 **(B)**, M3 is elaborated analogously to M2.

Mortar specimens are made in situ in accordance with the UNE-EN- 1015–11:2020 standard [28]. Three types of mortar are created to elaborate the experimental path outlined in this paper. One initial group of specimens generated by normal building mortar, referred to as M1, is made of Portland concrete. There is also a second group of modified mortar examples based on Portland concrete, referred to as M2 and M3. The above-mentioned ashes are used as a partial replacement for Portland concrete clinker (see Fig. 8), which acts as a binder in mortars M2 and M3. These ashes, which have undergone the various chemical and physical processes described above, replace 10% of the total mass of mortar binder (see Table 3) [44]. All specimens including M1, M2 and M3 are manufactured with same boundary conditions, securing the elimination of possible acquired differences during the hardening stage, for example the relative differences to environment or climate, because all specimens are situated in the same location. Furthermore, quantities of raw material, to elaborate the specimens, are the same with the exception of partial clinker substitution in M2 and M3 specimens. In the same way hardening period is done in the same boundary conditions of temperature and moisture. Using this experimental path, similarities and differences of the mechanical properties between M1, M2 and M3 specimens can be evaluated objectively. Equally the experimental mechanical tests, flexural and compression ones, are done in the same day and schedule, in order to respect experimental followed criteria of this paper [45].

**Table 3:** Composition of designed mortars in this paper.

Mortar Type	Composition			
	Sand [kg]	Concrete[kg]	Ashes[kg]	Water[kg]
**Standard M1**	4.5	1.5	0	0.9
**Modified M2**	4.5	1.35	0.15 (type I)	0.9
**Modified M3**	4.5	1.35	0.15 (type II)	0.9

**Fig. 9:**
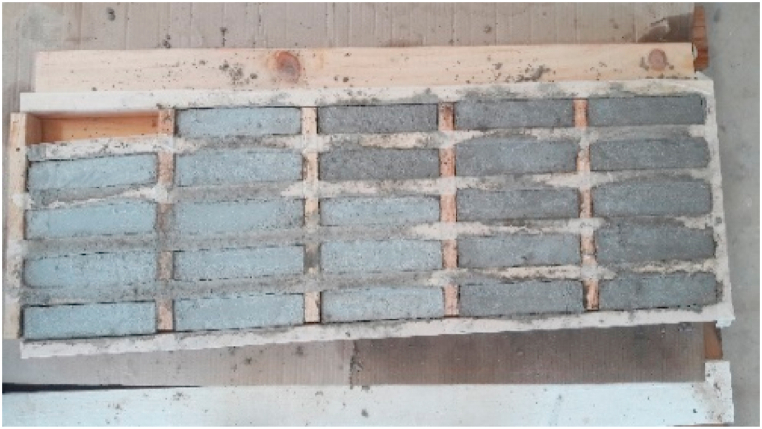
Production and molding of mortars.

There are 18 specimens required for the compositions described in Table 3. The prismatic specimens of 160 × 40 × 40 mm are manufactured in situ and classified into three groups, with six related to M1 mortar, six to M2, and six to M3. Due to the special needs of manufacture, a specific mold made of wood was created (See Fig. 9). In order to secure that specimens hardening period happen having the same boundary conditions of temperature, moisture and schedule. Securing that, in the moment of the breakage, all specimens are in the same stage of maturation of their mechanical properties.

Specimens manufacture process was developed under the specified environmental conditions on Table 4.

**Table 4:** Environmental conditions during the manufacturing process of the specimens.

Location	Portalegre District, Portugal
**Environment temperature**	26.3 °C
**Relative Humidity**	50%

The following table shows the environmental climatic conditions of the first 3 days that the specimens spent in the mold as UNE [27,28] standard specify (see Table 5).

**Table 5:** Environmental climatic conditions of the first 3 days of specimens.

	Average Temperature	Highest Temperature	Lowest Temperature	Average Relative Humidity
**Day 1**	26.1	30.2	22.2	50%
**Day 2**	21.5	22.5	20.7	59%
**Day 3**	19.9	23	15	63%
Specimens were placed outdoors out of the action of sun or rain.*				

**Fig. 10:**
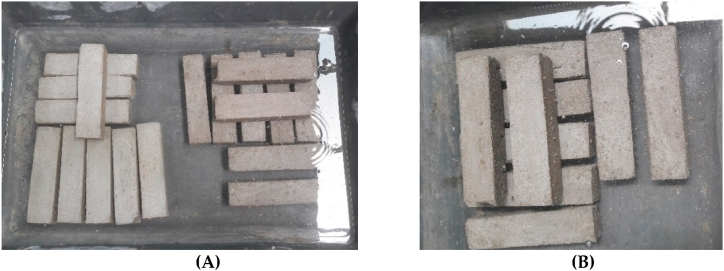
Curing (hardening) conditions of unmold specimens. M1 mortar specimens at left, M2 mortar specimens at right **(A)**. M3 mortar specimens **(B)**.

After the completion of the hardening process, which takes approximately three days, the specimens are taken out of the mold. Immediately after extraction, they are fully submerged in water at ambient temperature, as depicted in Fig. 10. The specimens remain in these identical boundary conditions until the execution of the experimental mechanical tests.

These breakage tests of the specimens are done at 7 and 28 days respecting experimental path of this paper abovementioned. Specimen's preparation steps are: at first, specimens are removed from water one day (24 h) before experimental mechanical tests, after, following day, possible impurities are removed from specimens, the surfaces of them and the test devices are cleaned with a clean cloth. Finally, to finish, specimens are tested with the devices dedicated to this purpose.

## 3 Results

**Fig. 11:**
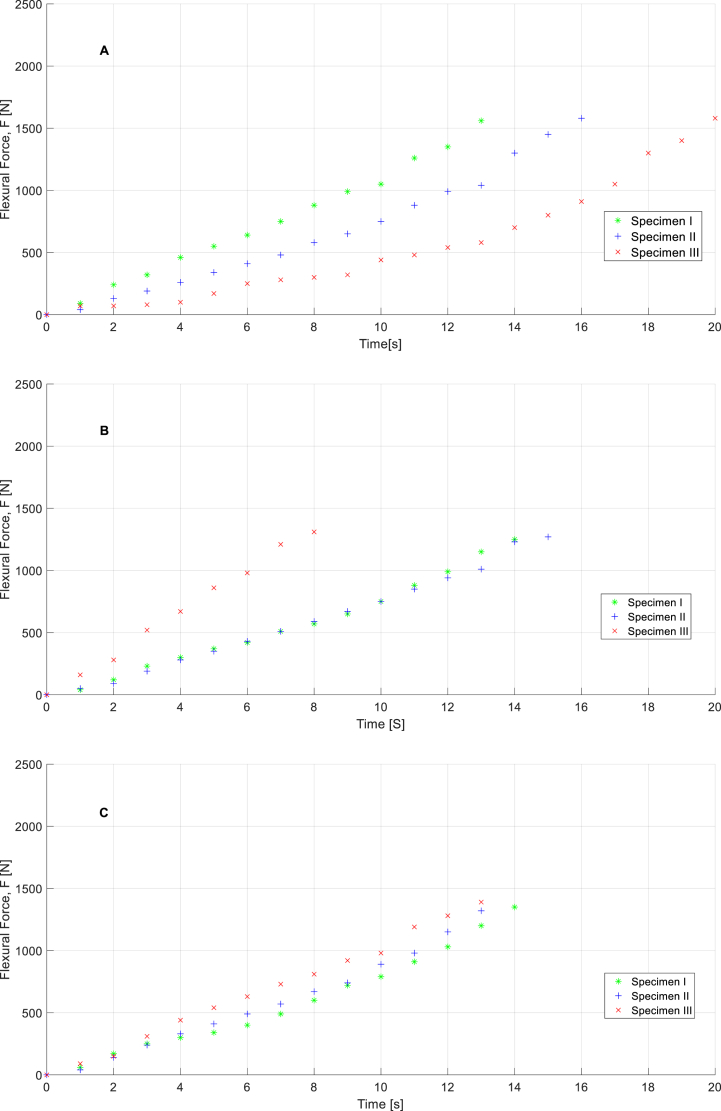
Curves for specimens related to flexural force vs time in case of M1 (**A**), M2 (**B**), and M3(**C**) in the time of 7 days.

**Fig. 12:**
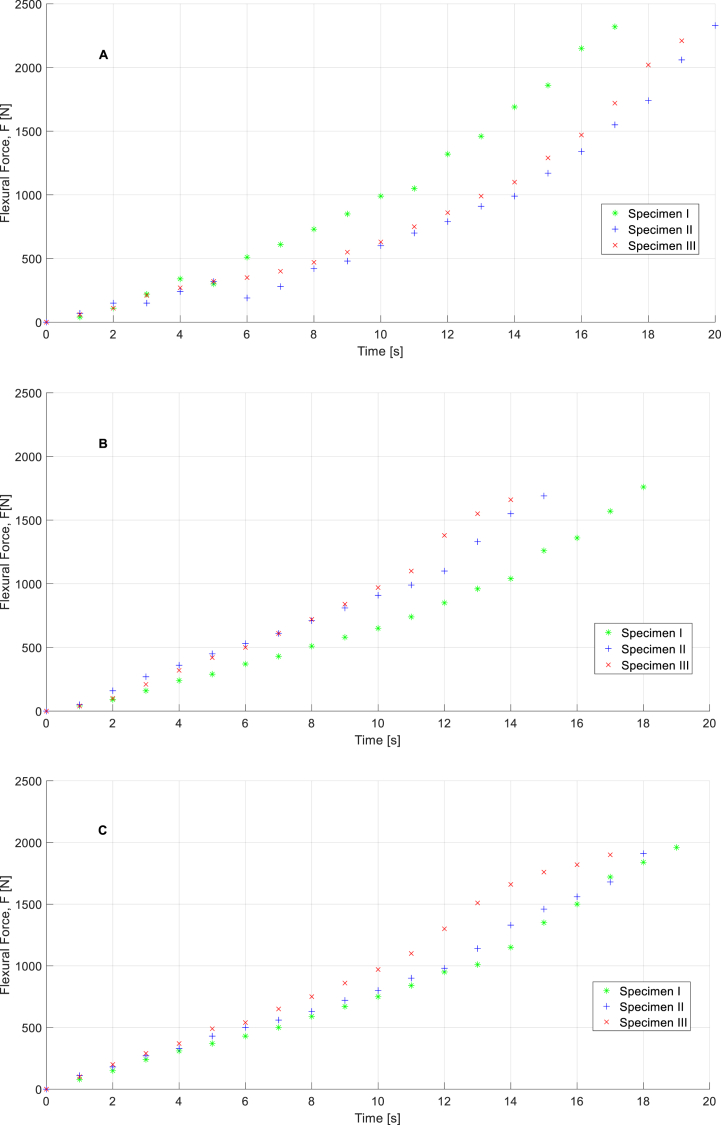
Curves for specimens related to flexural force vs time in case of M1 (**A**), M2 (**B**), and M3(**C**) in the time of 28 days.

**Fig. 13:**
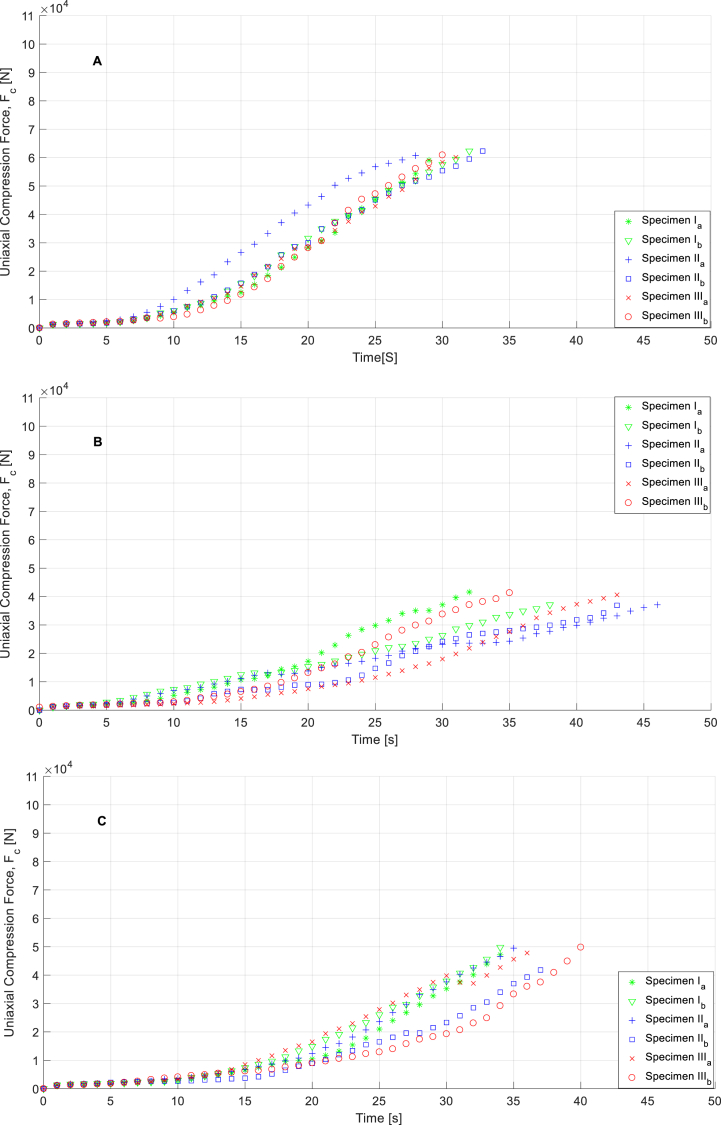
Curves for specimens related to uniaxial compression force vs time in case of M1 **(A)**, M2 **(B)**, and M3**(C)** in the time of 7 days.

**Fig. 14:**
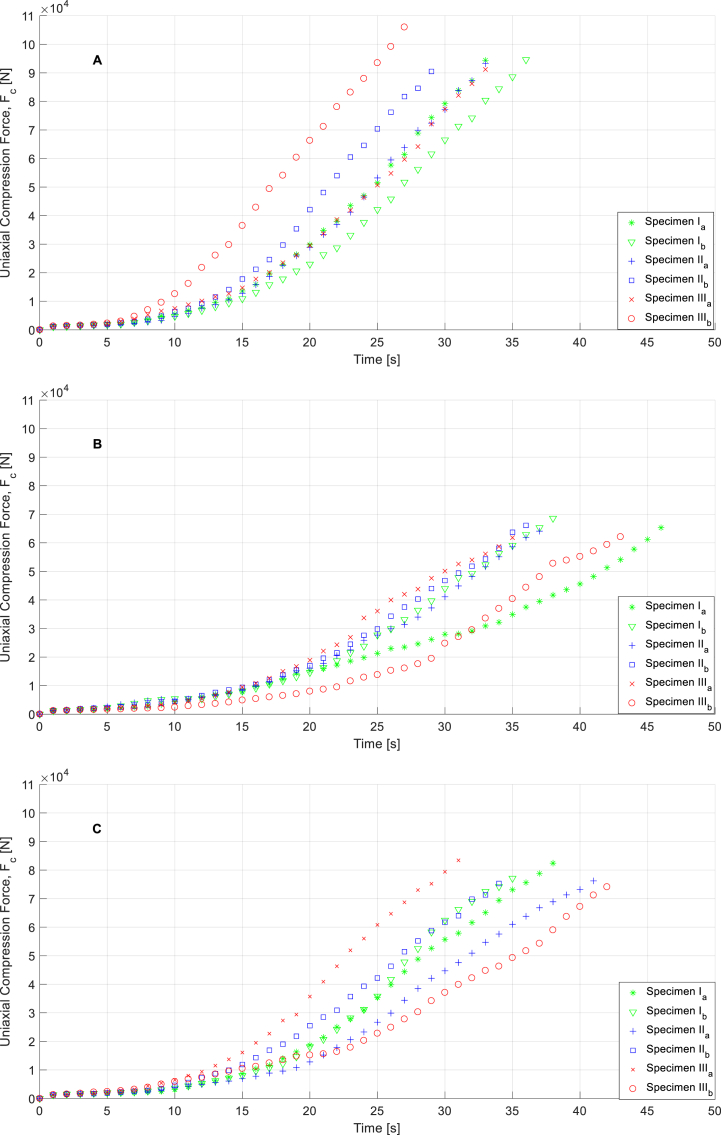
Curves for specimens related to uniaxial compression force vs time in case of M1 **(A),** M2 **(B)**, and M3 **(C)** in the time of 28 days.

Fig. 11a,b,c, Fig. 12a,b,c, Fig. 13a,b,c, and Fig. 14a,b, c show the experimental results obtained from the flexural strength and uniaxial compression forces over 7 and 28 days after executing the experimental mechanical test. These data include the entire test, from the beginning to the end, and demonstrate the maximum force that each specimen can withstand. Flexural stresses and uniaxial compression stresses can be determined from these experimental tests using the methodology described in the UNE-EN-1015–11:2020 [27] standard for flexural stresses and UNE-EN-196–1:2018 [28] standard for uniaxial compression stresses. (See Equations (1) and (2)).

(1) $$f=1,5\frac{F∙l}{b∙d^{2}}\text{;}$$

(2) $$R_{c}=\frac{F_{c}}{1,600}\text{;}$$

Where $f\left(N/{mm}^{2}\right)$ represents the flexural stresses on the specimens, F (N) is the load of flexural forces on the test specimens, l(mm) is the distance between of support pins, b(mm) and d(mm) are the dimensions related to cross area of the specimen (Due to all specimens share same size flexural stress tests associated parameters l, b, and d are considered constants whose values are 120 mm, 40 mm and 40 mm respectively). According to the second equation, $R_{c}\left(N/{mm}^{2}\right)$ represents the field values of the uniaxial compressive stresses, $F_{c}\left(N\right)$ represents the compressive load and 1600 is a constant obtained by calculating the cross-sectional area of the tested specimens $\left({mm}^{2}\right)$.

The specifications of the UNE-EN-196–1:2018 [28] standard establish that the test result is obtained by calculating the arithmetic mean of each of the three individual results. The result of each specimen is rounded to 0.05 N/(mm)^2^. The mean value of them is rounded to 0.1 N/(mm)^2^ in case of flexural stress calculations. To uniaxial compression stress calculations, standard specify that none of specimens, which are grouped in six packs, cannot individually present in its strength result variations greater than 10% in comparison with obtained arithmetic mean for the six group. If this condition is not verified, the result of uniaxial compression stress test taken is the arithmetic mean of the other 5 results. In case that two or more results among the six grouped specimens present variations greater than 10%, the entire test must be repeated.

Numerical results are expressed in Table 6, Table 7, Table 8, Table 9 of this section.

**Table 6:** Obtained results for flexural stress test for M1, M2 and M3 specimens in the time of 7 days.

Type	Specimen	Maximum Load [N]	f [$N/{mm}^{2}$]	Arithmetic Mean
**M1**	I	1,560	4.40	
	II	1,580	4.45	4.4
	III	1,580	4.45	
**M2**	I	1,250	3.50	
	II	1,270	3.55	3.6
	III	1,310	3.70	
**M3**	I	1,350	3.80	
	II	1,320	3.70	3.8
	III	1,390	3.90

**Table 7:** Obtained results for flexural stress test for M1, M2 and M3 specimens in the time of 28 days.

Type	Specimen	Maximum Load [N]	f [$N/{mm}^{2}$]	Arithmetic Mean
**M1**	I	2,320	6.55	
	II	2,330	6.55	6.4
	III	2,210	6.20	
**M2**	I	1,760	4.95	
	II	1,690	4.75	4.8
	III	1,660	4.65	
**M3**	I	1,960	5.50	
	II	1,910	5.35	5.4
	III	1,900	5.35

**Table 8:** Obtained results for uniaxial compression test for M1, M2 and M3 specimens in the time of 7 days.

Type	Specimen	Maximum Load[N]	$R_{c}\left[N/{mm}^{2}\right]$	UNE-EN-196–1:2018 Standard variation(%)	Arithmetic Mean $\left[N/{mm}^{2}\right]$
**M1**	I_a_	59,100	36.95	2.94	
	I_b_	62,300	38.95	2.31	
	II_a_	60,700	37.95	0.31	38.1
	II_b_	62,300	38.95	2.31	
	III_a_	60,100	37.55	1.36	
	III_b_	60,900	38.05	0.05	
**M2**	I_a_	41,600	26.00	6.38	
	I_b_	37,100	23.20	5.07	
	II_a_	37,100	23.20	5.07	24.4
	II_b_	36,900	23.05	5.68	
	III_a_	40,600	25.40	3.92	
	III_b_	41,300	25.80	5.56	
	I_a_	47,200	29.50	1.00	
	I_b_	49,800	31.15	4.53	
	II_a_	49,500	30.95	3.86	30.5
	II_b_	41,800	26.15	12.24*	
	III_a_	47,800	29.90	0.33	
	III_b_	49,800	31.15	4.53

**Table 9:** Obtained results for uniaxial compression test for M1, M2 and M3 specimens in the time of 28 days.

Type	Specimen	Maximum Load[N]	$R_{c}\left[N/{mm}^{2}\right]$	UNE-EN-196–1:2018 Standard variation(%)	Arithmetic Mean $\left[N/{mm}^{2}\right]$
**M1**	I_a_	94,400	59.00	0.60	
	I_b_	94,600	59.15	0.37	
	II_a_	93,200	58.25	1.88	58.0
	II_b_	90,500	56.55	4.74	
	III_a_	91,200	57.00	3.99	
	III_b_	106,000	66.25	11.58*	
**M2**	I_a_	65,300	40.80	0.94	
	I_b_	68,600	42.90	6.13	
	II_a_	64,100	40.05	0.91	40.4
	II_b_	66,100	41.30	2.17	
	III_a_	61,800	38.65	4.37	
	III_b_	62,100	38.80	4.00	
	I_a_	82,400	51.50	5.46	
	I_b_	77,100	48.20	1.29	
	II_a_	76,500	47.80	2.11	48.8
	II_b_	75,300	47.05	3.64	
	III_a_	83,400	52.15	6.78	
	III_b_	74,100	46.30	5.18	

As shown in Fig. 11a,b,c, Fig. 12a,b,c, Fig. 13a,b,c, Fig. 14a,b,c materials behaviors during flexural and compression mechanical tests are not identical. These differences are explained by the modification done in mortars compositions, topic which is evaluated in following sections of this paper.

## 4 Discussions

In this section of the manuscript is presented a comparison between mechanical characteristics and its progression along the hardening time. This comparison is done between same material manufactured specimens and specimens manufactured with different kind of ashes included on them. Comparing too, modified mortars M2 and M3 mechanical properties with the standard mortar based in Portland cement M1.

### 4.1 Mechanical properties progression of modified mortars

**Fig. 15:**
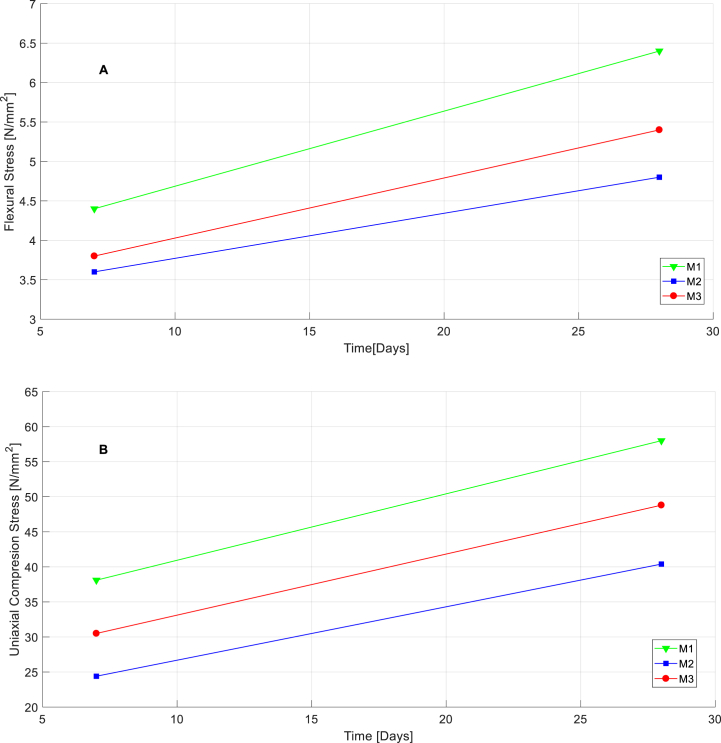
Curves of flexural strength (**A**) and compression strength (**B**) gain of mortars along 7 and 28 days.

Fig. 15a, b shows the progression of mechanical characteristics of the mortars during the cycle along 7 and 28 days.

Based in results obtained, it is shown that mechanical strengths during hardening stage of mortars along 28 days are analogous to the common standard for this kind of materials. Thus, for M1 reference mortar, 100% formed by CEM I 42.5 R of Portland clinker, mechanical strengths take the value 4.4 $N/{mm}^{2}$ for flexural strength and 38.1 $N/{mm}^{2}$ for uniaxial compressive strength. These values reach 6.4 $N/{mm}^{2}$ and 58.0 $N/{mm}^{2}$ after 28 days. As showing in Table 10, mechanical flexural strength acquired at first 7 days represent 68.75% of total strength. This result has a value of 65.69% in case of mechanical compressive strength.

**Table 10:** Flexural and compressive strength gains by mortars first 7 days.

Type	Flexural strength 7 days $\left[N/{mm}^{2}\right]$	Flexural strength 28 days $\left[N/{mm}^{2}\right]$	Flexural strength acquired first 7 days (%)	Compression strength 7 days $\left[N/{mm}^{2}\right]$	Compression strength 28 days $\left[N/{mm}^{2}\right]$	Compression strength acquired first 7 days (%)
**M1**	4.4	6.4	68.75	38.1	58.0	65.69
**M2**	3.6	4.8	75.00	24.4	40.4	60.39
**M3**	3.8	5.4	60.39	30.5	48.8	62.50

For M2, with 10% substitution of cement by biomass and WEEE, flexural mechanical strength at 7 first days is 3.6 $N/{mm}^{2}$ and 24.4 $N/{mm}^{2}$ for uniaxial compressive strength. These results go up to 4.8 $N/{mm}^{2}$ and 40.4 $N/{mm}^{2}$ respectively after 28 days. Thus, percentual flexural mechanical strength acquired first 7 days is 75.0%, 60.39% in terms of uniaxial compressive strength. As shown in Fig. 15a, graphically, trend of M2 is to describe a lower slope flexural mechanical strength curve that indicate an earlier growing of flexural capacities of this mortar, which represent a mechanical improvement respect M1 [46].

Finally, M3, with 10% substitution of cement by biomass, has a flexural mechanical strength of 3.6 $N/{mm}^{2}$ and 24.4 $N/{mm}^{2}$ to compressive at first 7 days. To 28 days, these values are 5.4 $N/{mm}^{2}$ and 48.8 $N/{mm}^{2}$ respectively. In numerical way in Table 10 and it is exposed during 7 first days M3 acquire 60.39% of total flexural mechanical strength and 62.50% in compressive terms.

In Fig. 15b, it shows that trend of percentual growing of mechanical uniaxial compression strength in mortars M2 and M3, in both cases, is similar to reference mortar M1.

### 4.2 Mechanical properties comparison between modified and reference mortars

In Table 11a and Table 11b are presented the comparisons between the results obtained in the experimental mechanical tests for M2 and M3 related M1 values. In numerical way, for the substitution of 10% of concrete clinker, mortar M3 presents loss of 15.86% of maximum compression strength in comparison with M1 and focusing in maximum flexural strength it loss 15.63% [47]. On the other hand modified mortar M2 presents loss of 30.34% in comparison with mortar M1, it means the losses are almost the double than M3, for the maximum compression strength. In case of the maximum flexural strength M3 presents losses of 25% in comparison with M1. It suppose losses around 10% bigger than modified mortar M3 [48].

**Table 11a:** Maximum flexural strength of modified mortars M2 and M3 in comparison with M1.

Type	Flexural strength first 7 days $\left[N/{mm}^{2}\right]$	Flexural strength comparison first 7 days (%)	Flexural strength for 28 days $\left[N/{mm}^{2}\right]$	Flexural strength comparison for 28 days (%)
**M1**	4.4	100.00	6.4	100.00
**M2**	3.6	81.81	4.8	75.00
**M3**	3.8	86.36	5.4	84.37

**Table 11b:** Maximum uniaxial compression strength of modified mortars M2 and M3 in comparison with M1.

Type	Uniaxial compression strength first 7 days $\left[N/{mm}^{2}\right]$	Uniaxial compression strength comparison first 7 days (%)	Uniaxial compression strength for 28 days $\left[N/{mm}^{2}\right]$	Uniaxial compression strength comparison for 28 days (%)
**M1**	38.1	100.00	58.0	100.00
**M2**	24.4	64.04	40.4	69.65
**M3**	30.5	80.05	48.8	84.14

During first 7 days, Flexural strength of M2 presents losses of 18.19% in comparison of M1 and for M3 losses are 13.64%. Talking about uniaxial compression strength M2 result is 35.96% lower than M1 maximum value in 7 days and M3 presents losses of 19.95%, this result is 16% bigger than M2 value in this case [49].

Taking strength values of M1 as reference is shown that mortar M3, which is modified with pine ashes only, presents an intermedium behavior showing 15.63% strength losses rate in case of M2, modified with a mixture of pine and WEEE ashes, these losses reach 30.34% in comparison with M1. However, behavior curves related with mechanical uniaxial compression strength of studied mortars (see Fig. 13, Fig. 14), which are done under the same boundary conditions to every specimen, shown M2 specimens present higher endurance in time terms in comparison with M3 specimens and this fact is even more evident in comparison with M1 specimens. It means M2 and M3 compounds, especially M2, present less fragile behavior that standard mortars. These characteristics open new favorable possibilities for some specific situations considering that fragile breakage or collapse breakage is one of the more relevant and dangerous in civil engineering.

The higher capacity of deformation of modified mortars present opportunities to substitute common standard mortars in those applications where the fragility of concrete carries some problems, not necessary under structural level, in cases like avoiding rift formations in finishes based on mortar or mortar-linings. Economically and in ecological terms the use of the modified mortars presented in this study has multiple advantages. Attending economic reasons, inclusion of ashes carries a lower production cost of mortar, this reduction numerically is proportional with the quantity of clinker based in Portland is replaced by ashes. Ecologically and in terms of recycling of resources the ashes obtaining process starts with the production of energy in form of syngas trough gasification process, after this the reuse of obtained waste is produced which are used as cost extenuating of raw material in mortar elaboration. In fact, this carry an elongation of the lifecycle of materials which form the components where WEEE are extracted to get the ashes which are gasificated mixed with biomass [50].

### 4.3 Mechanical properties comparison with other studies mortars

**Fig. 16:**
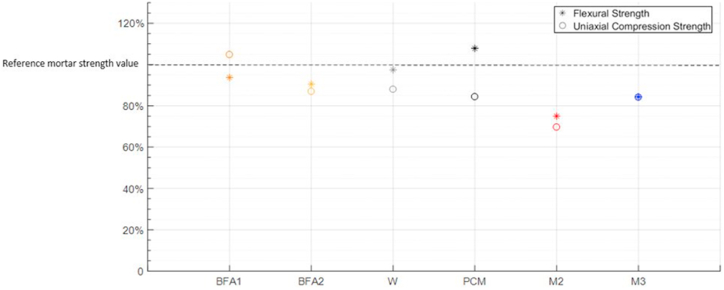
Flexural and uniaxial compression strength comparison among modified mortars in different.

In this section, mechanical strength results of M2 and M3 are compared with other modified mortars which already figure in the existing scientific literature. Comparison criteria for this final section of the manuscript must be precise to not induce errors in the comparative with other research works. So, for this comparison between mechanical behaviors are only selected modified mortars which were fabricated in analogous conditions to M2 and M3, above mentioned. Thus, to show a real comparative of mechanical properties between different researches about modified mortars, only those which 10% of clinker material have been substituted were selected and, furthermore, only those studies where flexural and compression strength results are present were selected. Already Verified both previous criteria, mechanical test done in selected manuscripts must be respect mortars hardening time, 28 days. This evaluation approach allows the comparison between the different mortars and its percentage variations in terms of gain or loss of strength after their modification. Comparation results are expressed in Fig. 16 in form of percentage. Where 100% strength value is the value, for flexural and compression strengths, obtained for the reference mortar of its own paper.

Scientific literature.

Where: BFA1 and BFA2 are modified mortars through a partial substitution of clinker by biomass fly ashes [51], W modified through a partial substitution of wollastonite [52], PCM mortar modification is a partial substitution of clinker through phase change materials [53]. M2 and M3 are the modified mortars obtained using the methodology expressed in this article.

## 5 Conclusions

The described methodology in this paper allows a double valorization and reuse of WEEE as manufacture components in mortars. The complete path of this methodology starts with the energy recovery of WEEE in form of gasification fuel. In a second step, by-products obtained through this thermo-chemical process have been included, by substitution of a clinker fraction, in construction mortars. Otherwise, ashes and slags which compound these by-products should be directly destined to landfill as inert. This approach of inert as by-products allows to include the ashes, through modified mortars creation, inside civil engineer field.

This research means a little advance to sustainability of existent planet resources and the recycle of endogenous and exogenous resources from a territory. Polymer materials lifespan is increased too, therefore, the pollution pressure over ecosystems due to these residues trend to decrease. Mechanical strength results of this ecological mortars are acceptable and allow its application in the civil engineer field in safety way despite the mechanical strength loss respect reference mortar.

Numerically, in case of M2 modified mortars, composed by Type I ashes (pine biomass and WEEE polymer) maximum value obtained to uniaxial compression strength is 40.4, so, the strength loss ratio, for a 10% substitution of clinker, is 30.35%. Analogously, for M3 modified mortars composed by Type II ashes (only pine biomass) maximum compression strength value registered is 48.80 N, it supposes a loss strength ratio of 15.86% in comparation with M1 (reference mortar). In case of maximum flexural strength values, M2 presents a loss ratio of 25%, and 15.63% in case of M3. Despite the fact exposed, is important to cite that even with loss strength registered due to partial Clinker substitution in modified mortars, maximum strength values are acceptable for some construction sector uses.

Future research areas in terms of modified mortars presents interesting topics related with civil engineering area. After the verification of higher flexibility presented by new modified specimens and chemical changes suffered due to the ashes inclusion on them, next steps in the study is a comparison treating other variables or different behaviors of modified mortars versus other external agents. Due to chemical composition and components included in mortars is predictable different responses of the material versus, for example, thermic insulation, acoustic insulation and permeability where this kind of modifications in mortars could replace in some cases to conventional insulators in cases of external protection of structural concrete or another elements in cases of mortar-finished. Finally, chemical alterations in modified mortars can confer beneficial properties in front of possible corrosion agents improving properties versus the oxidation or higher durability in high salinity environments.

To sum up, the mechanical results of the modified mortars in this study must be considered in a circular economy context and present a solution for the use and recovery of waste materials from the gasification of plastic materials which are able to be considered as materials to be valued and improved due to their polymeric nature, the insulating properties of modified mortars as well as their lightning and elastic properties in comparison with traditional mortars.

## Author contribution statement

Daniel Díaz-Perete, Manuel Jesús Hermoso-Orzáez, Julio Terrados-Cepeda: Conceived and designed the experiments; Performed the experiments; Analyzed and interpreted the data; Contributed reagents, materials, analysis tools or data; Wrote the paper. Pedro Silva-Romano: Performed the experiments; Analyzed and interpreted the data; Contributed reagents, materials, analysis tools or data. Cristina Martin Doñate: Analyzed and interpreted the data.

## Data availability statement

Data included in article/supplementary material/referenced in article.

## Funding

“This research received external funding. This work is funded by The Spanish Government's state-wide research project entitled: Sustainability and resilience of medium-sized cities and their contribution to the energy transition: circular urban metabolism, energy scenarios and indicator proposals. Acronym: METURBAN2030. Project type: Competitive R + D + I projects. Start date: January 12, 2022. End date: 11/30/2024. Funding entity: MINISTRY OF SCIENCE AND INNOVATION. GOVERNMENT OF SPAIN. Management centre: Research Management Service. Code: 2022/00407/001. Internal reference: MICIN_Transition_ECO_DIG_2021.”

## Declaration of competing interest

The authors declare that they have no known competing financial interests or personal relationships that could have appeared to influence the work reported in this paper.
